# Extravascular Implantable Cardioverter-Defibrillators: A Systematic Review of Emerging Evidence

**DOI:** 10.7759/cureus.85359

**Published:** 2025-06-04

**Authors:** Nikhil Jaganathan, Varun Goel, Robert Sorrentino, Dominic Gallo, Mallikarjuna Devarapalli

**Affiliations:** 1 Anesthesia and Perioperative Medicine, Augusta University Medical College of Georgia, Augusta, USA; 2 Cardiothoracic Surgery, Augusta University Medical College of Georgia, Augusta, USA

**Keywords:** anti-tachycardia pacing, clinical cardiac electrophysiology, extravascular-icd, implantable-cardioverter defibrillator, ventricular tachycardia (vt)

## Abstract

Implantable cardioverter-defibrillators (ICDs) are medical devices designed to prevent sudden cardiac death caused by life-threatening ventricular arrhythmias. This review aims to investigate the most novel approach via an extravascular lead by exploring preliminary data published in the PubMed, Cochrane, and ScienceDirect databases as of October 2024. An Oxford Level of Evidence was assigned to each paper. Papers rated Level 3B or 4 were included to ensure a high level of scientific rigor, including studies with conflicting findings. This benchmark was selected because the current literature does not include any systematic reviews of the subject (Level 1-3A), and Level 5 provides a poorly supported assessment. Of the 727 initial peer-reviewed records identified, 25 were included in the final analysis: 11 articles and 14 abstracts (including relevant literature regarding preclinical investigations of animal experiments). This data includes prospective trials and observational clinical studies, patient-reported outcome studies, subsequent analyses, and retrospective studies of efficacy, safety, and complications of extravascular ICDs (EV-ICDs) and therapeutic use. This review discusses the expectations from preclinical and clinical applications of EV-ICDs (extracted from both expert opinions and reported patient outcomes) and how certain limitations may be addressed based on the literature. A comprehensive review of complex medical devices is advantageous and enables multifaceted analysis of efficacy. This systematic review revealed that EV-ICDs demonstrate effective sensing, pacing, and defibrillation with high patient acceptance and low complication risk. In comparison with transvenous and subcutaneous ICDs, EV-ICDs provide effective antiarrhythmic action through antitachycardia pacing, safe lead removal, and enhanced long-term performance.

## Introduction and background

Implantable cardioverter-defibrillators (ICDs) are advanced medical devices designed to prevent sudden cardiac death caused by life-threatening ventricular arrhythmias. These devices monitor heart rhythms and deliver shocks to restore normal rhythm if dangerous arrhythmias occur. Traditional transvenous ICDs (TV-ICDs) are implanted with leads placed inside the heart through the venous system, which provide precise sensing and pacing capabilities [1]. However, these leads are prone to mechanical failures, battery depletion, and infections over time [1]. Subcutaneous ICDs (S-ICDs), an alternative innovation, are implanted beneath the skin, avoiding vascular structures [2]. While S-ICDs reduce lead-related complications, they lack the ability to provide pacing for bradycardia or antitachycardia pacing (ATP), an antiarrhythmic technique terminating ventricular tachycardias (VTs) with high efficacy [2]. Recent advancements, such as the Empower LP system and extravascular ICDs (EV-ICDs), aim to integrate pacing capabilities into non-transvenous systems, showing promising results in animal studies and early clinical trials [2]. The rationale for the development of EV-ICDs was focused on minimizing complications associated with TV-ICDs to optimize patient outcomes, especially in high-risk patients. The efficacy of both TV-ICDs and S-ICDs is well-established, with high rates of successful arrhythmia conversion and relatively low complication rates, though further research is needed to address the limitations presented by inappropriate shocks and pacing.

Although S-ICDs offered an improvement on TV-ICD technology, current research is aimed at addressing the limitations in bradycardia and ATP sensing via EV-ICDs. Similar to S-ICDs, EV-ICDs position the device outside the vascular system. In contrast to the subcutaneous site, however, the EV-ICD implant has leads commonly placed substernally in the anterior mediastinum or within the intercostal space [2]. This strategic placement allows the lead to lie directly above the ventricles, optimizing the sensing and defibrillation pathways while minimizing vascular and lead-related complications [2]. The substernal lead design typically includes shock coils and electrodes configured to provide effective defibrillation and pacing. During arrhythmia detection, the EV-ICD can deliver both high-energy shocks to terminate life-threatening arrhythmias and low-energy pacing to manage VT [3]. Early studies have demonstrated high success rates in defibrillation and pacing with fewer complications compared to transvenous systems, though implantation requires specialized training due to the anatomical positioning, complex procedural techniques, and potential risks such as pericardial or pleural space entry [3].

Differences exist between the EV-ICD and subcutaneous and transvenous ICDs. The former subcutaneous and transvenous ICD devices lack comparable lead reliability, present with complications attributed to vascular implantation, and lack ATP (in S-ICDs specifically) [4]. While TV-ICDs have demonstrated effective antiarrhythmic potential, vascular obstruction, disseminated infection, and lead malfunction present as serious postprocedural sequelae [4]. Moreover, the previous alternative, S-ICDs, only provide defibrillator capability, have a shortened battery lifespan, and have a substantial size compared to EV-ICDs [4]. The EV-ICD system utilizes substernal lead placement, ATP, and pause-prevention pacing to achieve antiarrhythmic outcomes similar to single-chamber TV-ICDs without intravascular or intracardiac hardware [5]. Through the use of a single substernal lead, this emerging device utilizes an ATP antiarrhythmic technique and reduces intraoperative complications, including cardiac and vascular damage, venous blockage, hemothorax, and pneumothorax [5]. EV-ICDs offer comparable battery life and size to TV-ICDs while potentially avoiding associated complications [6]. While the efficacy of transvenous and subcutaneous ICDs has been studied extensively in the past, the emerging technology of EV-ICDs has not been thoroughly studied. This systematic review aims to study the available data and provide a formative assessment of EV-ICDs to stimulate further investigation into their clinical application, including efficacy, safety, procedural aspects, and patient outcomes.

This article was previously posted to the Authorea preprint server on February 4th, 2025.

## Review

Methodology

Two authors (NJ, VG) conducted a systematic review of published manuscripts and abstracts, including prospective trials and clinical studies, subsequent analyses, and retrospective studies related to EV-ICDs, from PubMed, Cochrane, and ScienceDirect databases, from November 2014 to October 2024. Key words used in the search included “extravascular implantable cardioverter-defibrillator,” “EV-ICD,” and “substernal ICD.” Duplicate publications were excluded, and the remainder were analyzed based on their methodology, sample size, notable results, complications, and conclusions. Due to the limited nature of data available, published, peer-reviewed abstracts were included that met sufficient criteria of substance and level of evidence, as evaluated by the Oxford scale based on included methodological details [7]. Oxford scale adjudication was performed through independent verification of the quality of evidence with high inter-rater reliability. Included abstracts were required to report quantitative or qualitative procedural or patient outcomes. Non-English studies and studies with small sample sizes were not excluded due to the limited population of patients with the device; however, case reports were excluded. While some studies shared patient populations due to limited patient populations, differing analyses allowed for unique takeaways. In this systematic review, we highlight recent data regarding various topics related to EV-ICDs, including initial animal trials, intraprocedural challenges, long-term complications, antiarrhythmic performance (including sensing, pacing, and defibrillation), safety, lead removal, quality of life, and alternative lead implantation sites. Our discussion entails a thorough evaluation of the efficacy of EV-ICDs with comparison to S-ICDs and TV-ICDs, with comparison of procedural success, adverse reactions, and long-term safety. Ultimately, the objective of this systematic review is to provide an evidence-based approach to illustrate the use of EV-ICDs for VT and to delineate the efficacy and complications of the device.

Results

**Figure 1:**
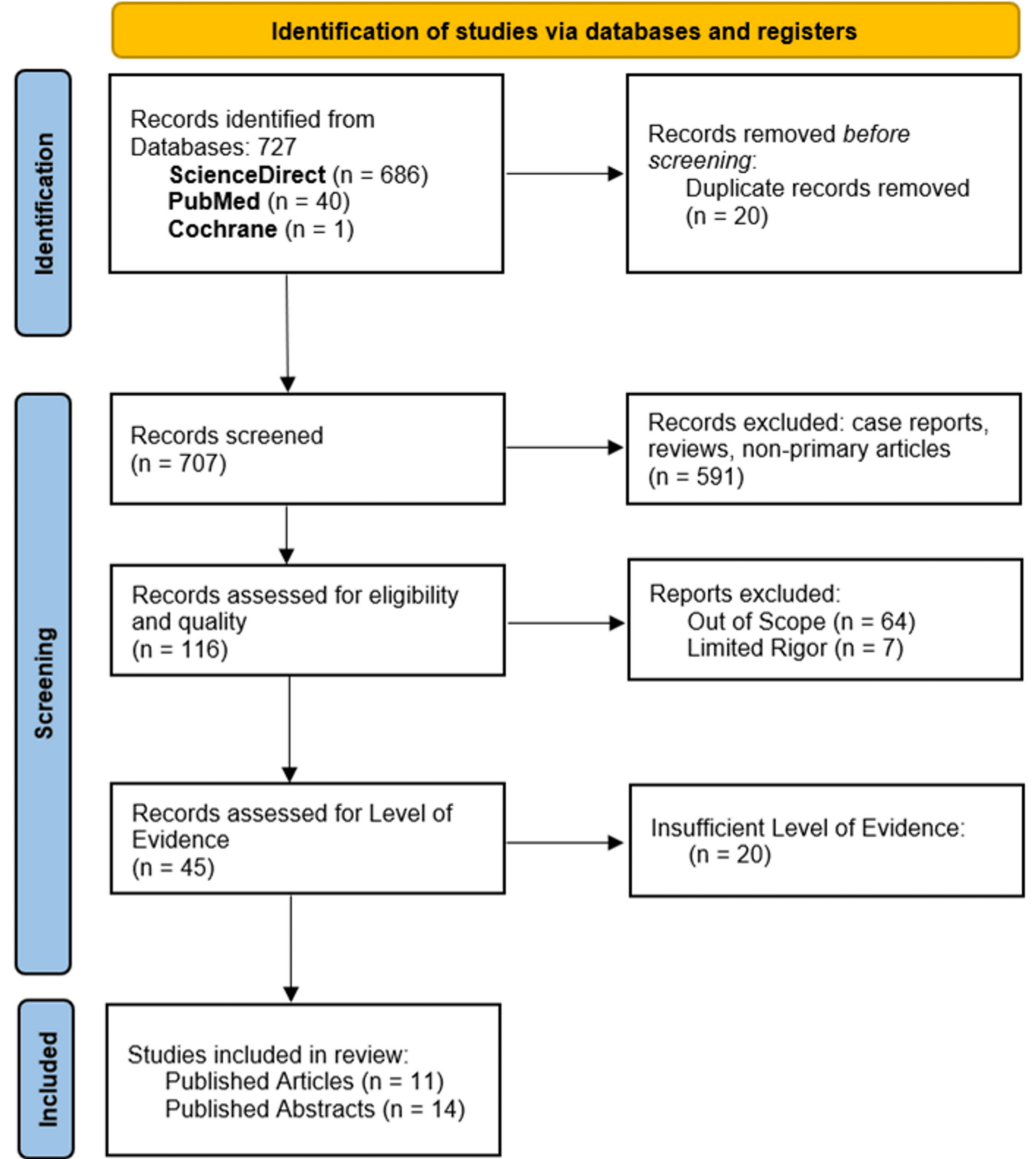
Preferred Reporting Items for Systematic Reviews and Meta-Analysis (PRISMA) flowchart showing study selection.

A total of 727 records were identified from the databases searched, of which 707 were designated for screening after removing duplicates. Following a review of the abstracts, 591 manuscripts were excluded due to either being case reports, reviews, or non-primary literature. Of note, there were no systematic reviews found in the literature regarding EV-ICDs. The remaining 116 manuscripts were reviewed and assessed for inclusion and exclusion criteria. Of these, 64 pertained to data outside of the scope of our systematic review, and seven had insufficient rigor, notably a lack of quantitative data. The level of rigor was evaluated based on study design and statistical analysis, while sample size was not solely utilized to ascertain insufficient rigor. Of the final 45 records, 25 met a sufficient level of evidence (Figure 1) [8]. There were 14 abstracts that included primary data, and 11 published articles. The data represented a vast record of studies on EV-ICDs, ranging from preliminary animal studies to multi-center large cohort studies.

There was a large variance among primary and secondary outcomes assessed by the included studies. Cumulatively, 10 studies included patient clinical outcomes, 19 included device performance, and 15 included findings regarding complications. Two studies were conducted in animal models to assess safety and efficacy. Four studies analyzed the incidence of infections in the implantation as well as the explantation of ICD leads. Six studies focused on a substernal location, which is the most common of the extravascular positions available. Two studies compared the safety and efficacy of EV-ICDs in direct comparison with either an S-ICD or a TV-ICD, with key comparison metrics including time to implantation as well as efficacy in detecting and terminating VT/ventricular fibrillation (VF) episodes. Four studies focused primarily on the application of pulse generators (PGs) in conjunction with EV-ICDs. Almost all of the records included in this study analyzed the ability of EV-ICDs to detect episodes of VF and VT as well as resolve them via ATP. Studies discussing comparisons between ICD models evaluated lead extraction ease and complications, quality of life and patient acceptance, infection rates, and other complications. The most significant disadvantage noted in most studies was the incidence of inappropriate shocks, commonly due to myopotential or P-wave oversensing (in up to 88% of cases), which can result in patient discomfort or subsequent bradycardia induced by ATP. Two studies discuss simulation via computing and in-silico models, which utilize patient data-driven algorithms to help identify oversensing to address this deficit in the future and enhance the safety and specificity of EV-ICDs.

Discussion

While the literature regarding EV-ICDs is continually emerging, several initial studies have assessed the safety, efficacy, and complications associated with these novel devices. Initial published studies explored the potential use of EV-ICD in animal models. The novel study by Thompson et al. represents the first long-term analysis of EV-ICD lead extractions. This study involved implantation of two EV-ICD leads and one transvenous lead in 24 adult sheep with annual evaluation of lead extractability and histology [9]. Although one episode of hemopericardium precipitated without cardiovascular injury during extraction, the study demonstrated safe removal of leads from the substernal space through either traction or simple extraction tools across a three-year duration [9]. An additional study conducted by Thompson et al. evaluated infections in 13 large animals receiving implantation of EV-ICD and transvenous resynchronization systems [10]. During the 12-18 weeks of follow-up, five infections were identified, including one infection related to the TV-ICD and four infections related to the EV-ICD [10]. While EV-ICD infections did not spread to the sternum or blood, the TV-ICD infection developed into endocarditis and sepsis, potentially highlighting the more consequential sequelae of TV-ICD infections compared to those of EV-ICDs [10]. These initial studies were conducted in animal models, which reduces the generalizability of the findings, yet they conveyed promising initial results regarding device safety. While these two animal studies explored a novel technology to assess potential extractability and establish the presence of complications such as infection, applicability to human models is limited, and small sample size prevents substantial comparisons between extravascular and transvenous ICDs [9,10].

Early studies examined the feasibility of extravascular pacing in a substernal location in human subjects. Brouwer et al. placed diagnostic pacing catheters in the substernal space of eight patients receiving midline sternotomy with analysis of unipolar and bipolar pacing configurations [11]. With ventricular capture established successfully in at least one configuration in five patients, the researchers concluded that substernal ventricular pacing in patients receiving midline sternotomy can be established [11]. Similarly, the Substernal Pacing Acute Clinical Evaluation (SPACE) study sought to assess the feasibility of pacing originating from substernal extravascular catheters implanted through a subxiphoid entry [12]. Across the 26 participants, successful catheter implantation was performed in all patients, achieving ventricular capture in 18 (69%) participants, with unsuccessful ventricular capture attributed to inappropriate positioning [12]. Initial studies such as these highlighted the efficacy and feasibility of extravascular catheter stimulation, which allowed this strategy to be incorporated in the EV-ICD. Brouwer et al. and the SPACE study investigated the feasibility of extravascular pacing through substernal catheters, providing fundamental early data about procedural and antiarrhythmic viability, yet these studies did not explore EV-ICD implantation in a clinical setting [11,12].

The initial Extravascular ICD Pilot Study by Crozier et al. was the first study evaluating substernal EV-ICD in humans and prospectively evaluated 21 patients across four sites with ICD referral, 20 of whom had EV-ICD implantation [4]. This pilot study demonstrated that the device effectively detected VF with at least 0.3 mV sensitivity across all patients [4]. Moreover, 95% of the patients demonstrated appropriate pacing under a maximum threshold of 10 V, and defibrillation was performed with 90% success [4]. The percentage of freedom from device-related systemic complications was 94.1%, with complications including inappropriate shock, inspiratory discomfort, minor wounds, and one episode of arrhythmogenic right ventricular dysplasia resolved with device removal [4]. Device removal was performed in five patients due to inadequate defibrillation testing in two patients, poor sensing in one patient, and lead dislodgement with inadequate defibrillation in two patients (elective removal) [4]. However, ultimately, the study demonstrated uncomplicated procedures with effective sensing, pacing, and defibrillation, highlighting the potential of the device for larger-scale studies [4]. Continued monitoring of this patient sample across three years post-implantation indicated stable functioning with appropriate sensing, pacing, and defibrillation based on pacing threshold and R-wave amplitude analysis [13]. Regarding complications, one patient demonstrated significant lead motion with a lack of pacing capture after six months, and two irregular shocks occurred due to environmental interference (6.9% yearly rate of inappropriate shocks) [12]. From three months to three years, the only reported ventricular arrhythmia was one patient receiving three shocks due to monomorphic VT, but chronic defibrillation testing indicated successful performance in all patients long-term [13]. The Extravascular ICD Pilot Study and its three-year monitoring represent early robust clinical data regarding EV-ICD implantation with quantitative evaluation of defibrillation, pacing, and sensing while exploring device-related and procedural complications and chronic evaluation of device stability. Although its sample size was limited, the Extravascular ICD Pilot Study facilitated the development of larger-scale EV-ICD trials [4,13]. Additionally, a follow-up analysis of the EV-ICD Pilot Study by Haqqani et al. highlights the minimally invasive nature utilized in the pilot study with leads inserted under the sternum through a para-xiphoid insertion site burrowed subcutaneously to the device in the left midaxillary position [14]. This analysis reported that three patients received device removal due to (1) lead tip movement causing irregular shocks, (2) elective removal due to lack of 10-J safety margin during defibrillation testing, and (3) persistent ATP-refractory VT [14]. However, no intraprocedural complications occurred during device removal, with simple traction alone necessary (no extraction tools), indicating safe explantation [14]. While the analysis reported by Haqqani et al. was qualitative in nature and highly limited in sample size, it evaluated important questions regarding device placement and reasons for removal [14].

The Extravascular ICD Pivotal study was a global prospective study conducted by Friedman et al., which included 356 patients with Class I or IIa indication of ICD, with implantation of EV-ICD attempted in 316 patients [5]. Overall, 98.7% of patients receiving induced ventricular arrhythmia had successful defibrillation, and 94.6% of patients had functional ICDs upon discharge [5]. Moreover, this study demonstrated no intraprocedural complications. Postoperatively, patients had 92.6% freedom from procedural and device-related complications six months postprocedurally, with major complications reported in 7.3% of patients, and 118 inappropriate shocks reported across 29 patients [5]. Moreover, long-term assessment of these patients demonstrated high chronic safety and efficacy with 91.9% freedom from complications, with the most common complication being lead dislodgement [6]. Across this analysis, approximately 50% of shocks were avoided due to ATP usage. The similar complication rate across 18 months, coupled with similar rates of inappropriate shocks (10.2% one year postprocedurally), suggests a lack of temporal decline in device functioning [6]. This study, with its significant sample size and prospective nature, demonstrated promising findings regarding the use of EV-ICDs for ventricular arrhythmias, including the pivotal role of ATP as an antiarrhythmic technique. The Extravascular ICD Pivotal Study yielded robust data over a broad patient population across multiple sites, allowing for multiple subsequent analyses. This study allowed for quantitative, evidence-driven conclusions regarding EV-ICD efficacy, complication risk, and long-term outcomes in patients with ICD implications, yielding numerous clinical implications [5,6].

One hypothesized advantage of the EV-ICD is the decreased occurrence of systemic infections compared to leads implanted within the vasculature, which was supported by the retrospective analysis conducted by Clementy et al. [15]. This analysis of the study by Friedman et al. demonstrated comparable infection incidence with subcutaneous lead implantation with an absence of systemic infection, including endocarditis, sepsis, and mediastinitis [15]. Across a mean 16.2 years of follow-up, 15 patients developed procedural or device-associated infection, necessitating device removal in six patients but primarily antibiotics and wound care [15]. The analysis by Clementy et al. yielded important clinical findings regarding the frequency of potential complications with important comparisons to S-ICDs [15]. With evidence suggesting reduced systemic infections with EV-ICDs compared to TV-ICDs, EV-ICDs may be preferred for infection-prone or immunocompromised patients who may be at greater risk with intravascular leads, but further study in these complex patient populations is necessary [15]. A retrospective analysis of lead removals during the EV-ICD Pilot, Pivotal, and Continued Access Studies by Sagi et al. involved 347 patients with completed EV-ICD implantation [16]. The analysis found that lead removal was performed adequately in 93.1% of cases, most often through simple traction without the need for extraction tools (the five patients with adhesions resulting from prolonged implantation times necessitated extraction sheaths). Moreover, no intraprocedural complications were observed during removal, and the 11 patients necessitating reimplantation had no complications [16]. Sagi et al. provided one of the largest EV-ICD analyses, allowing for a comprehensive, longitudinal evaluation of EV-ICD removals to establish safety [16]. Ultimately, these analyses indicate the low rates of systemic infection and safe lead removals in patients with EV-ICD implantation. Moreover, a prospective survey study of patients from the EV-ICD Pivotal Study conducted by Sears et al. involved participants completing the 12-Item Short Form Survey (SF-12) Quality of Life (QOL) survey at baseline and six months post-implant as well as the Florida Patient Acceptance Survey (FPAS) Quality of Life Survey six months post-implant [17]. Survey results demonstrated that SF-12 physical QOL improved significantly, and EV-ICD patients demonstrated greater FPAS acceptance of the device compared to subcutaneous or transvenous ICD patients [17]. Ultimately, this data demonstrates positive perceptions of the EV-ICD by patients, likely due to reduced discomfort or complications and improved body image associated with the less invasive device [17]. Sears et al. provided evidence substantiating EV-ICD outcomes through a novel lens: patient-perceived outcomes. Through validated surveys completed by patients, this study yielded important insights for consideration by future clinicians. Extrapolating this data may play an important role in the selection of antiarrhythmic devices for patients who cannot communicate their preferences [17]. This study has numerous impacts on clinical decision-making, as the prediction of patient quality of life and compliance with devices is fundamental for device effectiveness; thus, thorough patient-provider discussions of preferences and likelihood of acceptance are critical [17].

An additional analysis of the EV-ICD Pivotal Study database focusing on patients with prior S-ICD revealed safe, effective EV-ICD implantation in these patients with prior S-ICD status, having no effect on procedure time with improved patient acceptance, attributed by the authors to reduced device size and role of ATP [18]. While this analysis is highly limited in scope and sample size, it provides data for patient populations with prior S-ICD for consideration in future studies [18] In the retrospective analysis of sensing and detection efficacy in the Pivotal Study by Swerdlow et al., data regarding VF and spontaneous device-stored occurrences with adequate rate and duration in a programmed VT/VF therapy zone were examined [19]. This analysis demonstrated that EV-ICDs identified both spontaneous and induced ventricular arrhythmias appropriately, detecting all induced VF during implantation, with 95.9% of cases having a threefold safety margin; long-term efficacy was confirmed by the detection of all VT-VF episodes [19]. However, inappropriate shocks, a common complication, were most commonly caused by (1) P-wave oversensing and (2) myopotentials (electrical signals produced by muscular contractions) [19]. The former has been shown to be mitigated by use of a PWOS discriminator (statistically significant), calibration during implantation, or adjustment of lead placement. Swerdlow et al. and other numerical studies did not include quantitative comparisons regarding inappropriate shock rates between EV-ICDs, S-ICDs, and TV-ICDs, and a comparative analysis regarding this frequent complication is warranted. Swerdlow et al. performed robust quantitative analysis on EV-ICD Pivotal study data, importantly identifying frequent sources of inappropriate shocks. Due to the large, multicenter nature of this data, these study complications may have clinical correlations for future EV-ICD patients [19].

To assess the chronic performance of EV-ICD systems compared to traditional TV-ICD systems, Burke et al. conducted a non-randomized, single-center study with 18 patients who received both system implants. The EV-ICD system was placed in recording-only mode to compare sensing and detection without risk of therapy [20]. Of the 18 patients enrolled, 16 had a successful implant. Four patients exited the study due to complications, including pneumothorax and wound dehiscence, as well as an elective exit and an unrelated viral death [20]. Sensing and detection were assessed in 23 episodes total at 30-day and 90-day timepoints, which showed 100% sensitivity and detection by the EV-ICDs [20]. Burke et al. continued the analysis comparing the detection of cardiac episodes side-by-side with the TV-ICDs. Events were detected in seven patients, of whom two had three matched and appropriate detections. Among the remaining five patients with inappropriate event detection, the unmatched cases were due to background noise not detected by the TV-ICD and another due to diaphragm myopotential oversensing [21]. No other complications were reported in either analysis. These studies’ results indicate that the performance of EV-ICDs is not compromised at chronic checkpoints and remains a strong, viable alternative to TV-ICDs [21]. While this study conducted by Burke et al. is a strong tool for comparison, the EV-ICD systems implanted were not used therapeutically, and, as such, their true efficacy is not measured. Furthermore, the low sample size, single-center nature, and comparison with TV-ICDs but not S-ICDs due to the lack of available data impairs the comparative power and generalizability of the study.

The most common cause of inappropriate shocks in EV-ICDs is PWOS. To understand how to mitigate this, Swerdlow et al. designed a novel algorithm to be used by the EV-ICD sensor to reduce the amount of PWOS [22]. In the retrospective study, the MATLAB algorithm was applied to real data from 120 patients’ EV-ICD devices and correctly identified 43% of PWOS incidents, as well as 29% of non-cardiac oversensing episodes [22]. Despite the refined criteria for sensing, the algorithm did not incorrectly reject an episode of VF/VT as PWOS on any occasion, thereby maintaining full integrity of the device’s sensitivity [22]. While Swerdlow et al. provided evidence to support the use of software to reduce inappropriate therapy of devices, it must next be applied to a larger set of data from the Pivotal Study to refine the model before its use in clinically implanted devices.

O’Donnell et al. conducted a prospective, single-group, clinical study involving 26 patients, of whom 21 received EV-ICDs. Over two years, the study demonstrated stable pacing capture (5.1-5.3 V)and R-wave amplitudes (3.4-4.2 V), with the lowest amplitudes observed when patients lay on their right side in 17 patients [23]. Among notable outcomes, five episodes of spontaneous VT were successfully detected and resolved in one patient. This sensitivity and efficacy, albeit limited in scope, illustrate the device’s ability to detect and correctly deliver ATP in comparison to S-ICDs. While there were no intraprocedural complications, two patients experienced inappropriate shocks due to lead placement or oversensed electromagnetic interference, both of which are active areas of development for preventative measures. Additionally, nine adverse events were reported; three necessitated system removals due to one each of inappropriate shock, no safety margin, and intolerance to ATP [23]. The study concluded that EV-ICDs exhibited stable performance and a low incidence of adverse effects over the two-year follow-up period [23]. Although O’Donnell et al. performed one of the few long-term studies available, it was limited in its scope due to a limited sample size, and thus, these results only preliminarily indicate the longevity of these systems.

In contrast to ring electrodes, which set pacing via transmission of undirected current indiscriminately between cardiac and non-cardiac tissue, Burke et al. investigated the use of button electrodes, which preferentially target excitable myocytes [24]. The study assessed the level of sensation by 18 patients when the electrode was pacing at maximum voltage output and pulse widths, ranging from no sensation, sensation only, tolerable pain, or intolerable pain [24]. One patient was excluded from sensation testing due to atrial fibrillation with a rapid ventricular response, but of the remaining 17 patients, 24% reported no sensation, 59% reported sensation only, 12% reported tolerable pain, and one patient reported intolerable pain [24]. This latter case was reported as such when the patient was sitting upright, but reported sensation only when supine [24]. These results indicate that a button electrode may be a favorable design alternative to help improve patient discomfort outcomes. This investigation by Burke et al. prevents the risk of adverse effects from long-term therapy, but thereby limits its application of conclusions as pain sensation was subjectively reported by patients at maximum outputs rather than clinically relevant levels. Although this provides a framework for button electrodes to be explored as an alternative to ring electrodes in the future, additional studies would be necessary to assess therapeutic value in direct comparison.

Another study conducted by Breeman et al. investigated the lead placement time of a novel intercostal EV-ICD lead compared to substernal leads in 18 patients [25]. The study demonstrated that the novel intercostal EV-ICD lead could be implanted efficiently, with a 94% (16/17) success rate for attempted deployments and median times of 7.9 minutes for lead placement and 21.5 minutes for complete fixation and suturing of the site [25]. Two patients experienced challenges with deployment due to ossified cartilage or adhesions, but no major complications were noted [25]. This early experience suggests the procedure is predictable and compares favorably to other EV-ICD approaches, such as substernal leads, which average 35.5 minutes for complete fixation, though further data from multiple operators are needed [25]. This study, while limited by sample size, demonstrates a quantified, evidence-based comparison between substernal and extravascular approaches to ICD systems and offers valuable insights into surgical operation considerations.

Although a left lateral pocket position is typically employed for custom PG devices, Burke et al. investigated the safety and efficacy of a left pectoral position, which would allow commercially available PGs to be used in eligible patients [26]. Among 18 patients undergoing de novo ICD implantation or PG replacement, sensing and termination of induced VF episodes were compared against a control of the same patient using the left lateral device pocket [26]. When R-waves were above 1.0 mV, there was 100% sensitivity of induced VF episodes. In 83% of patients, the induced VF episode was terminated with a safety margin of 10 J, with one patient terminating with less than 10 J of safety margin [26]. The induced VF episode was unsuccessfully terminated in one patient with a body mass index (BMI) of 33.8 kg/m^2^; however, the same case was terminated from the left lateral position [26]. This failure was not investigated thoroughly in this experiment, but it invites further investigation into the role of obesity in EV-ICD efficacy. The findings suggest that the left pectoral region may be a feasible site for commercially available PGs, offering a cost-sensitive alternative. Burke et al. conducted this analysis and others from the same sample of patients, allowing a multifaceted inspection of outcomes. The investigation highlights the ability to pair this system with affordable options for patients, and can be replicated and expanded upon with more studies.

Burke et al. continued this investigation in another study with 36 patients to assess the safety and efficacy of a novel EV-ICD lead connected to commercially available DF-4 ICD PGs placed in the left mid-axillary and pectoral pockets [27]. All patients with the PG in the mid-axillary position had 100% defibrillation with less than 35 J, of which 89% defibrillated with greater than a 10 J safety margin. Among all patients, there was 100% sensitivity and resolution of induced VF episodes [27]. With no reported intraprocedural or postprocedural complications, the study indicates a high degree of fidelity with paired use of the novel EV-ICD lead with commercially available PGs [27]. In this follow-up investigation, Burke et al. address a concern of a narrow scope by increasing the sample size, although this is still limited in comparison with studies of larger magnitude and duration, such as the Pivotal Study. However, with similar conclusions reached as its predecessor, this study provides a reasonable basis for extrapolating results into clinical practice for patients who may require more affordable options.

To assess the safety and efficacy of PGs in a pectoral position, Chen et al. conducted an in-silico model simulation study, which was developed from the CT imaging of a patient with a parasternal EV-ICD lead and pectoral PG who completed defibrillation testing [28]. A total of 12 positions in the pectoral region were tested (four sub-muscular and eight subcutaneous implants) [28]. The simulations yielded data regarding the defibrillation threshold (DFT) in each model. The results indicated a DFT of 15.5 J achievable from the lateral aspect of the pectoral pocket, regarded as the optimal site, as this DFT is lower than in standard EV-ICD placements (20-30 J on average) [28]. Suboptimal sites resulted in DFTs with high, unpredictable variability dependent on precise implantation, identifying a potential challenge to achieving consistent results due to surgical implantation variance [28]. While there were no adverse events reported from any simulation results, the study indicated that the lateral aspect of the pectoral pocket remains most promising as a future alternative in conjunction with novel EV-ICD leads [28]. This study by Chen et al. applied similar principles from Burke et al. [26, 27] without risk of adverse events. This study offers a practical conclusion, but is limited to in-silico modeling and requires further testing in clinical settings.

Due to the potential impairment of defibrillation in patients with a higher BMI, Burke et al. investigated the efficacy of EV-ICDs in obese patients compared to non-obese patients [29]. Of the 36 patients enrolled in the study, 13 had a BMI greater than 30 kg/m^2^, indicated as a high BMI (HBMI), and were considered obese. The remaining 23 patients had a BMI lower than 30 kg/m^2^, referred to as low BMI (LBMI) [29]. Both groups displayed 100% sensing of induced VF episodes. Additionally, the mean intraoperative times for lead placement were measured to be 11.5 minutes for the LBMI group and 10.3 minutes for the HBMI group. Furthermore, the minimum energy required for defibrillation was almost identical across both groups [30]. Due to limitations in the sample size, the study was not conclusive in any differences between the cohorts in their associated rates of adverse events, though they reported that there were zero incidents in the HBMI group, and two (9%) in the LBMI group [30]. This analysis by Burke et al. addressed a notable concern of patients who are at risk of cardiovascular disease and accidents. The quantitative measures definitively indicated that patients with elevated BMIs maintain this therapeutic option, although further studies of patient populations with BMIs greater than 40 kg/m^2^ may be warranted to investigate the impact of body habitus on long-term outcomes and safety profiles.

Fundamentally, key literature-driven clinical benefits of EV-ICDs include strong patient acceptance, ATP efficacy with successful defibrillation, and reduced complication risk. Clinical performance demonstrates that substernal EV-ICDs have similar longevity, defibrillation level, and size as TV-ICDs [31]. Mean battery longevity for EV-ICDs has been demonstrated to be 11.8 years compared to 7.3 years for S-ICDs, allowing for reduced frequency of replacement [32]. These features, combined with an extravascular implantation, have contributed to positive impacts on patient quality of life, but real-world implementation is necessary for more comprehensive evaluation of device performance.

The Risk of Bias in Non-randomized Studies-of Interventions, Version 2 (ROBINS-I V2) scale was utilized to assess risk of bias, since all 25 studies were nonrandomized studies evaluating interventions with similar or overlapping patient populations [33]. Each of the 25 included studies were evaluated for the seven bias domains included in ROBINS-I, namely, bias due to confounding, bias in selection of participants into the study, bias in classification of interventions, bias due to deviations from intended interventions, bias due to missing data, bias in measurement of outcomes, and bias in selection of the reported result [33]. Four studies were identified as having a “Serious” risk of bias. Of these, two were due to being animal studies with insufficient detail regarding what criteria animal subjects were selected; two were due to a very low sample size. Bias analysis of all other studies demonstrated “Moderate” or “Low” risk of bias across the seven ROBINS-I domains, with adequate assessment of outcomes and high scientific validity. The most common risk domains to indicate “Moderate” risk of bias were risk due to confounding and bias in the selection of reported outcomes. This was most commonly due to the lower sample sizes present in the studies, as well as a limited use of statistical analysis or thorough outcome tracing. This assessment was first made independently by two authors (NJ, VG) and discussed with another author (MD) to resolve discrepancies. Table 1 provides the risk of bias designations of the included studies.

**Table 1:** Risk of bias assessment for included studies based on ROBINS-1 V2 scale. Domains of risk assessment: D1: bias due to confounding; D2: bias due to selection of participants; D3: bias in classification of interventions; D4: bias due to deviations from intended interventions; D5: bias due to missing data; D6: bias in measurement of outcomes; D7: bias in selection of the reported result [33]. ROBINS-1 V2: Risk of Bias in Non-randomized Studies-of Interventions, Version 2

Risk of bias domains								
Study	D1	D2	D3	D4	D5	D6	D7	Overall
Crozier et al. (2020) [4]	Moderate	Moderate	Low	Low	Low	Moderate	Moderate	Moderate
Friedman et al. (2022) [5]	Moderate	Moderate	Low	Low	Moderate	Low	Moderate	Moderate
Friedman et al. (2023) [6]	Moderate	Moderate	Low	Low	Moderate	Low	Moderate	Moderate
Thompson et al. (2022) [9]	Moderate	Serious	Moderate	Low	Moderate	Low	Moderate	Serious
Thompson et al. (2023) [10]	Moderate	Serious	Moderate	Moderate	Moderate	Moderate	Moderate	Serious
Brouwer et al. (2017) [11]	Moderate	Moderate	Low	Low	Moderate	Low	Moderate	Moderate
Sholevar et al. (2018) [12]	Moderate	Moderate	Low	Low	Moderate	Low	Moderate	Moderate
Crozier et al. (2023) [13]	Moderate	Moderate	Low	Low	Low	Moderate	Moderate	Moderate
Haqqani et al. (2021) [14]	Serious	Serious	Low	Low	Moderate	Moderate	Moderate	Serious
Clementy et al. (2024) [15]	Moderate	Low	Low	Low	Moderate	Moderate	Moderate	Moderate
Sagi et al. (2024) [16]	Moderate	Moderate	Low	Low	Moderate	Low	Moderate	Moderate
Sears et al. (2024) [17]	Moderate	Moderate	Low	Low	Moderate	Moderate	Moderate	Moderate
Amin et al. (2023) [18]	Moderate	Moderate	Low	Low	Moderate	Low	Moderate	Moderate
Swerdlow et al. (2024) [19]	Moderate	Low	Low	Low	Moderate	Low	Moderate	Moderate
Burke et al. (2023) [20]	Moderate	Low	Moderate	Low	Moderate	Low	Moderate	Moderate
Burke et al. (2023) [21]	Moderate	Low	Moderate	Low	Moderate	Low	Moderate	Moderate
Swerdlow, et al. (2023) [22]	Moderate	Low	Moderate	Low	Low	Low	Moderate	Moderate
O’Donnell, et al. (2021) [23]	Moderate	Low	Moderate	Low	Moderate	Low	Moderate	Moderate
Burke et al. (2024) [24]	Moderate	Low	Moderate	Low	Low	Moderate	Moderate	Moderate
Breeman et al. (2023) [25]	Moderate	Low	Moderate	Low	Low	Low	Moderate	Moderate
Burke et al. (2024) [26]	Moderate	Low	Moderate	Low	Low	Low	Moderate	Moderate
Burke et al. (2023) [27]	Moderate	Low	Moderate	Low	Low	Low	Moderate	Moderate
Chen et al. (2024) [28]	Moderate	Low	Serious	Low	Low	Low	Moderate	Serious
Burke, et al. (2023) [29]	Moderate	Low	Moderate	Low	Low	Low	Moderate	Moderate
Burke et al. (2023) [30]	Moderate	Low	Moderate	Low	Low	Low	Moderate	Moderate

Table 2 presents a summary of the methodology, sample size, notable results, complications, and conclusions from the included studies.

**Table 2:** Summary of the methodology, sample size, notable results, complications, and conclusions of included studies. A qualitative assessment of published articles and abstracts regarding EV-ICDs. EV-ICDs: extravascular implantable cardioverter-defibrillators; TV-ICDs: transvenous implantable cardioverter-defibrillators; ATP: antitachycardia pacing; VT: ventricular tachycardia; VF: ventricular fibrillation; PGs: pulse generators; HBMI: high body mass index; LBMI: low body mass index

Study	Methodology	Sample size	Notable results	Complications	Conclusion summary
Crozier et al. (2020) [4]	Prospective, non-randomized multi-center pilot study	21 patients	20 patients had satisfactory implantation. 90% of the patients were at a safety threshold of at least 10 J during defibrillation testing. Pacing was 95% effective at a maximum of 10 V. Median defibrillation threshold (15 J), mean ventricular fibrillation amplitude (2.8 mV), and mean R-wave amplitude (3.4 J) were appropriate	No complications during implantation. Explantation of the device was performed in two patients following discharge	As the first human EV-ICD trial, this study demonstrated significant practicality and efficacy of sensing, pacing, and defibrillation with substernal EV-ICD leads
Friedman et al. (2022) [5]	Prospective global nonrandomized, single-group clinical study	356 patients, including 316 patients with an EV-ICD implantation attempt	98.7% of patients with induced ventricular arrhythmia had successful defibrillation, and 94.6% of patients were discharged with a functional ICD. 92.6% of patients were free from procedural or device-associated complications six months postprocedurally	No significant intraprocedural complications occurred. 29 patients received 118 inappropriate shocks for 81 arrhythmic episodes. 25 major complications were recorded six months postprocedurally in 23 patients	EV-ICDs were successfully implanted and effectively identified and eliminated induced ventricular arrhythmias
Friedman et al. (2023) [6]	Prospective global non-randomized, single-group clinical study (follow-up from the Pivotal Study [5])	316 patients with an EV-ICD implantation attempt	91.9% of patients had freedom from procedural or device-associated complications 18 months postprocedurally. Through 18 months, 19 patients underwent 80 episodes of arrhythmia effectively treated by shock (28 episodes), ATP (37 episodes), or ATP and shock (15 episodes). All episodes treated with shock were terminated, while 32 out of 52 episodes were terminated effectively by ATP	Lead dislodgement was the predominant complication, occurring 10 times in 9 patients within 120 days of implantation. 10.2% rate of inappropriate shocks (in 35 patients)	Long-term evaluation of EV-ICD systems demonstrated safety and efficacy with ATP-mediated shock avoidance in approximately 50% of spontaneous arrhythmias
Thompson et al. (2022) [9]	Prospective non-randomized single-group animal study	24 adult sheep	Compared lead removal using traction alone vs. extraction tools over three years. One year: five EV-ICD lead removals (two with traction alone); no TV leads removed. Two years: no leads removed with traction alone. Three years: one of eight EV-ICD leads and two of four TV leads removed through traction	One hemopericardium with tamponade was reported (did not result in cardiovascular injury with extraction). Two inversions of the ventricle with reduced cardiac output due to transvenous leads	First chronic extraction of EV-ICD leads demonstrates safe removal from the substernal space through traction and simple tools across a three-year duration in sheep
Thompson et al. (2023) [10]	Prospective non-randomized single-group animal study	13 large animals (ovine, porcine, canine) receiving implanted EV-ICD and transvenous resynchronization systems	Across the 12–18 weeks of follow-up cultures, five infections were detected (including one TV-ICD-related infection and four EV-ICD-related infections). Two EV-ICD infections were managed with system removal and antibiotics, while two EV-ICD infections were managed with antibiotics alone	No advancement of EV-ICD infections to the sternum or hematogenous spread. In contrast, the TV-ICD developed into endocarditis and sepsis	Infections associated with EV-ICD systems can be managed with antibiotics and device explantation
Brouwer et al. (2017) [11]	Prospective non-randomized study	Eight patients receiving midline sternotomy	Sternotomy patients received a diagnostic pacing catheter in the substernal space with analysis of various unipolar and bipolar pacing strategies and strength-duration curves to determine optimal output. Ventricular capture was established in at least one configuration in five patients. The 60-mm electrode spacing configuration yielded the most effective bipolar configuration. Unipolar pacing was effectively established in three out of four patients	NA	Substernal ventricular pacing in patients receiving midline sternotomy is achievable
Sholevar et al. (2018) [12]	Prospective non-randomized study	26 patients undergoing substernal electrophysiology catheter implantation	Successful catheter implantation in all patients (11.7-minute mean procedure duration). Ventricular capture was achieved in 18 patients (69%)	Unsuccessful ventricular capture was attributed to inappropriate positioning. Decreased stimulation was found in one patient	Substernal extravascular pacing is feasible, highlighting the potential for extravascular pacing devices
Crozier et al. (2018) [13]	Follow-up of a pilot study [4]	14 patients receiving follow-up for three years after implantation	Six patients received defibrillation with at least a 10 J safety threshold. Long-term defibrillation testing was appropriate in all patients (success ≤40 J). Prior to three years, device removal was performed in three patients with traction and no extraction tools. The only reported ventricular arrhythmia was one patient who received three shocks due to monomorphic ventricular tachycardia	For three years, while seven patients had stable lead placement or minimal movement, one patient demonstrated significant lead motion with lack of pacing capture after six months. One patient experienced deflection of the lead tip with reduced effectiveness, resulting in elective device removal, while another had removal due to ATP-refractory VT. Two irregular shocks occurred due to environmental artifacts (6.9% yearly rate of inappropriate shocks)	Pacing threshold and R-wave amplitude data across three years demonstrate effective chronic performance of the EV-ICD with stable sensing, pacing, and defibrillation
Haqqani et al. (2021) [14]	Follow-up analysis of a pilot study [4]	21 patients who underwent EV-ICD implantation as a part of the EV-ICD pilot study	Three patients received device removal due to (1) lead tip movement causing irregular shocks, (2) elective removal due to lack of 10-Joule safety margin during defibrillation testing, and (3) persistent ATP-refractory VT. Intact lead removals were performed via simple traction from the substernal space	No intraprocedural complications during device removal	Substernal EV-ICD lead removal is safe during the 310 days post-implantation without complications
Clementy et al. (2024) [15]	Retrospective analysis of a prospective global non-randomized, single-group clinical study (Pivotal Study [5])	316 patients with an EV-ICD implantation attempt	No reports of endocarditis, sepsis, or mediastinitis with no systemic infection, indicating similar infection incidence as with subcutaneous ICD	15 patients developed procedural or device-associated infection across a mean of 16.2 months, including eight major, three minor, and four observational complications. These complications were managed with antibiotics +/- wound care with ICD removal in six patients	Comparable infection incidence was demonstrated in the initial EV-ICD Pivotal Study as the rates reported with the use of subcutaneous ICD. Among the patients in this analysis, an absence of endocarditis, sepsis, and mediastinitis was noted
Sagi et al. (2024) [16]	Retrospective analysis of lead removal experience during EV-ICD Pilot, Pivotal, and Continued Access Studies	347 patients with EV-ICD implantation (from the three original studies)	Lead removal was performed in 8.4% of patients, most commonly for lead dislodgement (mean implantation time of 12.6 months). Complete lead removal was satisfactory in 93.1% of cases. While 84.6% of removals utilized simple traction, 15.4% of removals involved extraction tools	No intraprocedural complications were noted during removal, and the 11 device reimplantation procedures were uncomplicated	Across the first three years following implantation, substernal lead removal is feasible and safe
Sears et al. (2024) [17]	Prospective completion of the 12-Item Short Form Survey (SF-12) Quality of Life (QOL) survey at baseline and six months post-implant, as well as the Florida Patient Acceptance Survey (FPAS) Quality of Life Survey six months post-implant (follow-up from the Pivotal Study [5])	247 patients who were included in the EV-ICD Pivotal Study	SF-12 physical QOL improved significantly, while SF-12 mental QOL did not improve significantly. Sex, ICD shock experience, and atrial fibrillation did not have a statistically significant difference. EV-ICD patients demonstrated greater FPAS acceptance of the device compared to subcutaneous or TV-ICD patients (based on historical data)	NA	Quantified patient-reported quality of life data at baseline and six months indicate improved physical QOL and acceptance of the device in comparison to transvenous and subcutaneous ICD
Amin et al. (2023) [18]	Retrospective analysis of the Pivotal Study [5] data of patients with previous subcutaneous ICD implantation	Nine patients of the EV-ICD Pivotal Study who had received prior subcutaneous ICD implantation (with secondary EV-ICD implantation)	The average time between subcutaneous ICD removal and EV-ICD implant was approximately 35 days. EV-ICD implantation duration was similar among patients with prior subcutaneous ICD (79 minutes) and without prior subcutaneous ICD (60.5 minutes)	No significant intraprocedural complications, only one significant follow-up complication (infection at device site)	EV-ICD can be safely implanted in patients with prior subcutaneous ICD implantation with no impact on procedure time among patients with prior subcutaneous ICD. Use of ATP and reduced size may contribute to the high acceptance rate of EV-ICD compared to subcutaneous ICD
Swerdlow et al. (2024) [19]	Retrospective analysis of sensing/detection efficacy based on Pivotal Study [5] EV-ICD patients	299 patients of the EV-ICD Pivotal Study	All EV-ICDs identified induced VF during implantation. EV-ICDs successfully detected VF in 95.9% of cases with a threefold safety margin. Follow-up testing revealed that EV-ICDs detected all 59 VT/VF episodes. Oversensing contributed to 87.9% of non-VT/VF occurrences, while supraventricular tachycardia contributed to 12.1%	Inappropriate shocks were common, most predominantly due to P-wave oversensing (PWOS) (which is decreased by utilizing a PWOS discriminator and calibrating sensing during implantation). Oversensing was primarily caused by myopotentials (61.2%) and PWOS (19.9%), but inappropriate shocks were only delivered in 3.2% of myopotentials and 21.8% of PWOS occurrences	EV-ICDs identified both spontaneous and induced ventricular arrhythmias appropriately
Burke et al. (2023) [20]	Non-randomized, single-center study	18 patients implanted with TV-ICD and EV-ICD in recording mode	16/18 successful placements. Sensing and defibrillation were appropriate in 100% of cases	Four patients exited the study due to pneumothorax, wound dehiscence, elective removal, and unrelated viral death. One patient in acute heart failure required 35 J maximum output	At 90 days follow-up, the EV-ICD was effective at sensing and detecting VF episodes
Burke et al. (2023) [21]	Non-randomized single-center study	18 patients implanted with TV-ICD and EV-ICD in recording mode	16/18 successful placements. Spontaneous tachycardia was detected in seven patients. Two patients had three matched appropriate events	Two patients had unmatched inappropriate events due to a broken anode cable and diaphragm myopotential oversensing	A left, anterior parasternal EV-ICD has comparable sensing of cardiac rhythms with PGs compared to TV-ICDs
Swerdlow et al. (2023) [22]	Statistical modeling and simulation	MATLAB testing of an algorithm using real data from 120 patients with an EV-ICD implant	The algorithm was able to identify 43% of PWOS and 29% of non-cardiac oversensing	The algorithm incorrectly rejected episodes of VF/VT zero times (no compromise on sensitivity)	A novel algorithm to address PWOS, the most common cause of inappropriate shocks, is able to reduce PWOS and non-cardiac oversensing by significant amounts with no compromise to detecting VT/VF
O’Donnell et al. (2021) [23]	Prospective single-group clinical study	26 patients with 21 implanted	Five episodes of spontaneous VT were detected and resolved successfully in one patient. Pacing capture and R-wave amplitudes are stable over time and lowest with the patient lying on the right side relative to lying on the left or prone position	No intraprocedural complications. Two patients received an inappropriate shock due to either lead placement or oversensed EMI. Nine adverse events in two years, with three resulting in system removals	Two-year follow-up of patients with EV-ICD indicates low rates of adverse effects and stable results
Burke et al. (2024) [24]	Prospective, single-center study	18 patients at pre-discharge visit	No sensation (24%), sensation only (59%), tolerable pain (12%), intolerable pain (6%). Occurred only in the sitting posture, with the same patient reporting sensation only when supine. Pain levels never prevented pacing capture threshold testing	One patient was excluded from sensation testing due to atrial fibrillation with rapid ventricular response	Novel intercostal EV-ICD lead produced low levels of patient discomfort during pacing at maximum settings
Breeman et al. (2023) [25]	Non-randomized, single-center study	18 patients with intercostal lead sutured to the fascial plane	Lead deployment was successful in 94% (16/17) of attempted procedures. Median time of 7.9 minutes for lead placement and 21.5 minutes for complete fixation	One patient could not undergo lead deployment due to ossified costal cartilage obstructing the intercostal space. In another patient, adhesions prevented adequate lead deployment, resulting in removal without testing. No additional complications were reported	The novel intercostal lead is not only faster but also reliable in results compared to existing substernal leads
Burke et al. (2024) [26]	Prospective, non-randomized paired analysis involving induced VF testing in patients undergoing de novo ICD implantation or PG replacement	18 patients with either de-novo implantation or PG replacement	Sensing of induced VF episodes from pectoral position was 100% in patients with R-wave greater than 1 mV, and unsuccessful in one patient with R wave of 0.3 mV. Termination of VF was successful in 83% (15/18) with a 10 J safety margin, and in 11% (2/18) with a <10 J safety margin. Unsuccessful in one patient with HBMI from the pec pocket, but successful with margin from the lateral pocket	No significant complications. One patient failed to terminate the VF episode from the left pectoral PG position but was successful from the left lateral pocket	The efficacy of pulse generation from the pectoral position versus the lateral is comparable and safe
Burke et al. (2023) [27]	Non-randomized, single-center, acute study	36 patients with de novo or replacement EV-ICD	Successful defibrillation using ≤35 J in 100% of patients with left mid-axillary PG placement and 83% with left pectoral PG placement. All episodes sensed, detected, and shocked	No serious device-related intraoperative adverse events were reported	Demonstrated success and efficacy of EV-ICDs with PGs on the market from both mid-axillary and left pectoral positions
Chen et al. (2024) [28]	In-silico model based on CT imaging	12 different pectoral PG positions were simulated	A defibrillation threshold of 15.5 J was achievable with the PG in the optimal lateral aspect of the pectoral pocket. Suboptimal PG positions produced a wide range of DFTs, with outcomes highly dependent on precise placement	The study did not report any patient complications or adverse events but highlighted potential challenges with DFT variability due to suboptimal PG positioning	Intercostal EV-ICD lead could be paired with a pectoral PG. Defibrillation success depends heavily on precise PG placement, with optimal outcomes achieved in the lateral pectoral pocket
Burke et al. (2023) [29]	Retrospective analysis of a prospective global non-randomized, single-group clinical study	36 patients with implants -23 <30 BMI -13 >30 BMI	Sensing of induced VF was 100% in both groups. Time to lead placement and the lowest energy required for defibrillation were similar across both groups	There were no serious adverse events in the HBMI group, and an insignificant amount (9%) in the LBMI group	Efficacy of an EV-ICD is comparable between BMI groups, with similar energy required for defibrillation and no compromise to VF sensitivity
Burke et al. (2023) [30]	Follow-up analysis of [27]	36 patients with implants -23 <30 BMI -13 >30 BMI	21/23 successful placements in LBMI with 11.5-minute mean lead placement time. 12/12 in the HBMI group with a mean time of 10.3 minutes	No adverse effects	There is no significant difficulty in implementing an EV-ICD with obese patients

## Conclusions

EV-ICDs have undergone multifaceted studies in consideration of numerous variables. EV-ICDs first demonstrated safer lead removals in animals compared to TV-ICDs. Completion of initial studies that demonstrated the feasibility of extravascular substernal pacing with subxiphoid entry highlighted the capacity for substernal implantation and attributed unsuccessful ventricular capture to inappropriate positioning. Larger-scale studies have substantiated the effective sensing, pacing, and defibrillation of ventricular arrhythmia with low device-related complications, such as inappropriate shock, minor wounds, inspiratory discomfort, lead dislodgement, or inadequate sensing/defibrillation. Subsequent analyses demonstrated continued efficacy, no intraprocedural complications for device removal, successful defibrillation, high freedom from complications, low complications in obese patients, and effective ATP antiarrhythmic technique. Although rare, the predominant complications include lead dislodgement and inappropriate shocks due to PWOS and myopotentials, while TV-ICDs demonstrate more severe complication risks, such as infection. Moreover, compared to TV-ICDs and S-ICDs, EV-ICDs demonstrate improved long-term outcomes and patient perceptions. Additional analyses demonstrated comparable infection incidence with subcutaneous lead implantation, with an absence of systemic infection, safe lead removal (most often without extraction tools), and improved quality of life and patient acceptance. Ultimately, the most critical clinical implications of EV-ICD technology include ATP effectiveness, favorable defibrillation outcomes, low complication risk, and high patient satisfaction. As this field continues to advance, clinical studies will grow in both size and frequency. From the limited set available currently, it is difficult to confidently ascertain whether or not EV-ICD systems will be the future of preventing sudden cardiac death. To address this limitation, larger-scale studies should be conducted, such as the Extravascular ICD Pivotal study, which can gather data from more patients in a wider range of settings. Additionally, the non-randomized nature of most existing literature introduces a moderate risk of bias, thereby limiting the ability to significantly interpret results. Aspects necessitating further study include effectiveness in high-risk patient populations and real-world performance in diverse groups. Moreover, more tailored research is warranted regarding the use of button electrodes and alternative EV-ICD implantation sites, including the efficacy of intercostal compared to substernal leads. However, given the current data available, early indications of this emerging field remain encouraging for millions of patients. With initial data demonstrating safe implantation, explantation, and arrhythmia termination using ATP, EV-ICDs may facilitate effective antiarrhythmic management within select patient populations in future clinical practice.
